# Electrochemical monitoring of enzymatic cleavage in nanochannels with nanoparticle-based enhancement: determination of MMP-9 biomarker

**DOI:** 10.1007/s00604-023-05835-7

**Published:** 2023-06-12

**Authors:** David Valero-Calvo, Celia Toyos-Rodriguez, Francisco Javier García-Alonso, Alfredo de la Escosura-Muñiz

**Affiliations:** 1 NanoBioAnalysis Group, Department of Physical and Analytical Chemistry, University of Oviedo, Julián Claveria 8, 33006 Oviedo, Spain; 2 Biotechnology Institute of Asturias, University of Oviedo, Santiago Gascon Building, 33006 Oviedo, Spain; 3 NanoBioAnalysis Group, Department of Organic and Inorganic Chemistry, University of Oviedo, Julián Clavería 8, 33006 Oviedo, Spain

**Keywords:** Nanopores, Nanochannels, Nanoparticles, Electrochemical, MMP-9, Enzymatic cleavage

## Abstract

For the first time the use of nanoparticles as carriers of an enzymatic substrate immobilized inside nanoporous alumina membranes is proposed with the aim of amplifying the nanochannel blocking produced and, consequently, improving the efficiency of an enzyme determination through enzymatic cleavage. Streptavidin-modified polystyrene nanoparticles (PSNPs) are proposed as carrier agents, contributing to the steric and the electrostatic blockage due to the charge they present at different pH values. Electrostatic blockage is the predominant effect that governs the blockage in the interior of the nanochannel and is dependent not just in the charge inside the channel, but also in the polarity of the redox indicator used. Hence, the effect of using negatively charged ([Fe(CN)_6_]^4−^) and positively charged ([Ru(NH_3_)_6_]^3+^) redox indicator ions is studied for the first time. Under the optimum conditions, matrix-metalloproteinase 9 (MMP-9) is detected at clinically relevant levels (100–1200 ng/mL) showing a detection limit of 75 ng/mL and a quantification limit of 251 ng/mL with good reproducibility (RSD: 8%) and selectivity, also showing an excellent performance in real samples with acceptable recovery percentages (in the range around 80–110%). Overall, our approach represents a cheap and fast sensing methodology of great potential in point-of-care diagnostics.

**Graphical Abstract:**

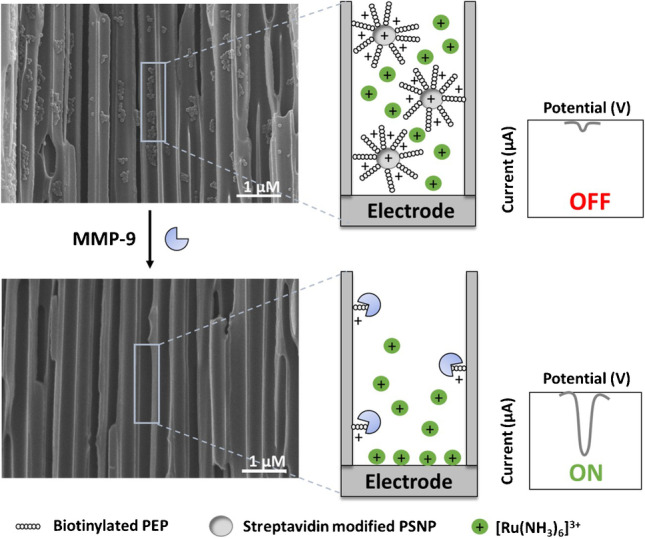

**Supplementary Information:**

The online version contains supplementary material available at 10.1007/s00604-023-05835-7.

## Introduction

Nanopore/nanochannel-based sensing consists in the alteration of an ionic flow through a nanopore by the presence of single analytes that cross it under the application of an external potential [1]. This principle has been long applied to the detection of single molecules, such as DNA, RNA, peptides, or proteins, constituting the so-called stochastic sensing [2]. Although biological nanopores were first used for sensing applications, solid-state nanoporous membranes have emerged as an alternative, providing their enhance stability and easiness for sensing large molecules [3].

Nanoporous alumina membranes stand out among such solid-state materials, due to their specific structural properties (narrow pore size and a high pore density (1·10^9^/cm^2^)), easy functionalization, low unspecificity, and mass production availability [4], and have been long applied in optical [5, 6] and electrochemical [7, 10] sensing. Typically, the sensing principle consists in the immobilization in the inner walls of the nanochannels of a bioreceptor (antibody, aptamer, or DNA) that specifically recognizes the analyte of interest. Once the analyte is retained inside the nanochannel, it blocks the passage of a redox indicator solution through the membrane to an electrode, due to both size (steric) and charge (electrostatic) effects [11, 13] what is commonly monitored through voltammetric measurements.

However, the use of nanoporous membranes for monitoring enzymatic cleavage processes has been scarcely explored so far [9], as the small size of the enzymatic substrates does not produce a significant nanochannel blocking.

In this context, we propose for the first time the use of nanoparticles as carriers of an enzymatic substrate to overcome this drawback, as they contribute to amplifying the blocking produced and, consequently, to improving the efficiency of the enzyme determination.

Streptavidin-modified polystyrene nanoparticles (PSNPs) are proposed as carrier agents, applied to the detection of matrix-metalloproteinase 9 (MMP-9), a potential biomarker overexpressed in different diseases, including cancer (breast, ovarian, cell lung, colorectal cancer) [14], neurodegenerative diseases [15, 16], or chronic wounds [17]. MMP-9 is a gelatinase enzyme that presents specific cleavage activity over the peptide substrate Leu-Gly-Arg-Met-Gly-Leu-Pro-Gly-Lys (PEP) [18], so in our approach, this peptide is conjugated with the PSNP for MMP-9 sensing.

The incorporation of nanoparticles amplifies the blocking produced both sterically and electrostatically, as they introduce a high charge density inside the channel. Such charges depend on the pH of the measurement buffer, so a careful study of this parameter is shown in this work. Moreover, the interaction with the charges generated inside the nanochannel and the polar redox indicators used for the voltammetric measurements highly determines the blockage obtained. Negatively charged ([Fe(CN)_6_]^4−^) and positively charged ([Ru(NH_3_)_6_]^3+^) redox indicator ions have been evaluated in this work, a pioneer study of key relevance for this kind of sensing systems, which may be extended for the detection of other analytes.

## Experimental

### Chemicals and equipment

N-(3-Dimethylaminopropyl)-N′-ethylcarbodiimide hydrochloride (EDC), N-hydroxysulfosuccinimide sodium salt (sulfo-NHS), (3-aminopropyl) triethoxysilane (APTES), phosphate-buffered saline (PBS) tablet pH 7.4, bovine serum albumin (BSA), peptidoglycan from *Bacillus* subtilis, MMP-9 pre-activated human, lysozyme from chicken egg white, uric acid, hexaammineruthenium(III) chloride [Ru(NH_3_)_6_]Cl_3_, and potassium ferrocyanide K_4_[Fe(CN)_6_] were purchased from Sigma-Aldrich (Spain). Peptide substrate Leu-Gly-Arg-Met-Gly-Leu-Pro-Gly-Lys (PEP) with biotin in N-Terminal (98.9% purity) was purchased from Abyntek (Spain). PSNPs with a nominal size of 0.09 µm (0.1% w/v) were purchased from Spherotech (USA). Human MMP-9 enzyme-linked immunosorbent assay (ELISA) kit was obtained from Abcam (UK). Redox indicator media used consisted of 10 mM K_4_[Fe(CN)_6_] and 500 µM [Ru(NH_3_)_6_]Cl_3_ solutions prepared in different buffers: 0.1 M sodium acetate (NaAc) pH 4.6 and 0.1 M Tris (tris(hydroxymethyl) aminomethane)-HCl (Tris–HCl) pH 7.2. The buffer solution 2-(N-morpholino ethanesulfonic acid) (MES) at 0.1 M pH 5 was also prepared. All solutions were prepared in ultrapure water with 18.2 MΩ cm resistivity 25 °C (Millipore Direct-Q® 3 UV from Millipore Iberica S.A. (Spain)).

For the spike and recovery assay, a fresh saliva sample was collected from a healthy donor in a polypropylene tube as described in Supplementary information.

Nanoporous alumina membranes (Whatman™, Anodisc™ filters, 60 µm thickness, 13 mm diameter, and 100 or 200 nm pore sizes) were purchased from VWR Avantor® (Spain). Indium tin oxide (ITO)–coated polyethylene terephthalate (PET) film with surface resistivity of 60 Ω/sq was purchased from Sigma-Aldrich (Spain) and cut in pieces of 4.3 cm × 2 cm defining an 8 mm diameter working electrode. Counter and reference electrodes were made of platinum wire (Alfa Aesar; USA) and silver/silver chloride (CH Instruments, Inc.; USA) respectively. All the electrochemical measurements were performed with an Autolab PGSTAT 128N from Methrom (Switzerland), connected to a computer and controlled by Nova 2.1.4 software.

For the bioconjugation, a MSC-100 Thermo-Shaker from Labolan (Spain) and a Rotanta 460R thermostatic centrifuge from Hettich (Germany) were employed. Streptavidin PSNPs and the final bioconjugate of polystyrene nanoparticles/biotinylated peptide (PSNPs/PEP) were characterized by transmission electron microscopy (TEM) using a JEM-1011 operated at 100 kV from JEOL (Japan), by dynamic light scattering (DLS-ζ potential) using a Zetasizer Nano SZ Malvern Instruments (UK) and by Fourier transform infrared spectroscopy (FTIR) using a Varian 620-IR from Agilent (USA). Nanoporous alumina membranes without and with the bioconjugate were examined by scanning electron microscopy (SEM) using a JSM 6610LV from JEOL (Japan) with an accelerating voltage of 20 kV. For the incubation steps, a MIR-262 SANYO incubator from SANYO Electric Co. Ltd. (Japan) was used.

### Methods

#### Conjugation of PSNP with biotinylated PEP

PSNPs were conjugated with the biotinylated PEP following the procedure provided by Spherotech with slight modifications [19]. Briefly, 500 µL of commercial streptavidin-modified PSNP (0.1% w/v) were incubated with 10 µL of PEP (0.01 mg·mL^−1^) at 650 rpm, 25 °C for 30 min using a Thermo-Shaker incubator. Then, the PSNP/PEP conjugate was centrifuged at 7500 rpm (6100 g), 4 °C for 40 min, and washed twice with 10 mM PBS pH 7.4. Finally, the obtained PSNP/PEP conjugate was re-suspended in 500 μL of 10 mM PBS pH 7.4.

#### Immobilization of the PSNP/PEP conjugate and enzymatic cleavage by MMP-9

Nanoporous alumina membranes of 200 nm pore size (see the optimization of the nanopore size in Supplementary information) were first functionalized with amine groups using a well-established methodology [20]. The detailed process is described in Supplementary information and schematized in Fig. S1. Then, 30 µL of a mixture of PSNP/PEP solution containing 5 mM EDC/sulfo-NHS was added to the membrane and left incubating overnight at 4 °C (see the incubation time optimization in Supplementary information). Afterwards, membranes were washed three times with 10 mM Tris–HCl pH 7.2.

Enzymatic cleavage of the PSNP/PEP conjugate was performed by placing 30 µL of the MMP-9 solution (prepared at different concentrations in 10 mM Tris–HCl pH 7.2) in the membrane and incubating for 2 h at 37 °C, as this temperature is optimal for favoring the catalytic activity of MMP-9 [21]. Finally, membranes were thoroughly washed with the measurement buffer used in each case before measuring. For the control assays, the same procedure was followed but instead of the PSNP/PEP conjugate, either a 10 mM PBS pH 7.4 solution or a non-conjugated peptide solution (in 10 mM PBS pH 7.4 buffer containing 5 mM EDC/sulfo-NHS) was added to the membranes.

Selectivity studies were conducted following the same experimental procedure but replacing MMP-9 with 600 ng·mL^−1^ solutions of UA and Lys at concentrations of 250 μM and 50 μg·mL^−1^, respectively, as they correspond to the range in which they are present in saliva samples [22, 23]. The selected interference analytes were evaluated alone and in combination with MMP-9. Additionally, other analytes of interest as BSA or peptidoglycan were evaluated at a concentration of 600 ng·mL^−1^ to test the suitability of the developed sensor in other matrixes.

#### Spike and recovery assay

Spike and recovery protocol was performed to study the MMP-9 detection in the saliva sample matrix of healthy donors. Saliva samples were collected as explained in the Supplementary information and spiked for a final concentration of 200, 300, and 600 ng·mL^−1^ of MMP-9 respectively. Samples were analyzed, without any pre-treatment, by the addition of 30 μL of spiked sample to each membrane. Later, membranes were thoroughly washed with 0.1 M NaAc pH 4.6 (6 times instead of 3 times due to the viscosity of the sample) and measured following the protocol described below. Finally, the cathodic peak current obtained for the reduction of [Ru(NH_3_)_6_]^3+^ at approximately − 0.20 V was taken as analytical signal, and the % of recovery in the saliva sample between this current and the one obtained using standard samples in Tris–HCl buffer. The initial concentration of MMP-9 in the selected samples was evaluated using a commercial ELISA kit.

#### Electrochemical measurements

Electrochemical measurements were performed using an electrochemical cell set-up widely established in our research group [24]. A detailed description is given in Supplementary information, together with illustrative pictures (Fig. S2).

For the measurements, 400 µL of a redox indicator solution (prepared in buffers at different pH) was added to the electrochemical cell. [Ru(NH_3_)_6_]^3+^ was used as a positively charged redox indicator at a concentration of 500 µM and measured by differential pulse voltammetry (DPV) (details in Supplementary information), recording the reduction of [Ru(NH_3_)_6_]^3+^ ions to [Ru(NH_3_)_6_]^2+^ at approx. − 0.20 V as analytical signal. [Fe(CN)_6_]^4−^ was used as a negatively charged redox indicator at a concentration of 10 mM, again using DPV (details in Supplementary information), taking the oxidation of [Fe(CN)_6_]^4−^ ions to [Fe(CN)_6_]^3−^ at + 0.40 V as analytical signal.

To study the long-term stability of the PSNP/PEP-modified membranes, nanochannel blocking was evaluated on different days after storing them at 4 °C for 3 weeks.

## Results and discussion

### Nanochannels blocking by PSNP/PEP conjugate: electrostatic effects involved

As stated before, PSNP are used in this work as carriers of the peptide immobilized in the nanochannels, with the aim of maximizing their blocking, and the later unblocking by the enzymatic cleavage action of the MMP-9 analyte. Prior to their use, PSNP/PEP conjugate was characterized (detailed in Supplementary information), showing a reduced nanoparticle polydispersity, and confirming PEP successful immobilization.

The nanochannel blocking was evaluated using redox indicator ions that flow through the channels to reach the electrode, where they were voltammetrically monitored. It is important to note that the flux of the redox indicator ions through the nanochannels is strongly influenced by electrostatic effects, which are related to the charge of the molecules immobilized in their inner walls as well as to the charge of the indicator ions themselves.

Therefore, a careful study of such key phenomena using both positively charged ([Ru(NH_3_)_6_]^3+^) and negatively charged ([Fe(CN)_6_]^4−^) redox indicator ions was evaluated. Buffer solution media at different pHs were used, since pH is a key parameter determining the charge of the molecules immobilized in the inner walls of the nanochannels. The theoretical isoelectric point (pI) of the target peptide is 11 (see Fig. S3 in Supplementary information) while the streptavidin protein that coats the PSNP has an isoelectric point of 5–6 [25].

The analytical signals obtained for the different redox indicators and pHs were compared, in terms of the intensity of the analytical signal recorded, for bare membranes and for membranes modified with PEP and with the PSNP/PEP conjugate.

The analytical signals obtained for the different redox indicators and pHs were compared, in terms of the percentage change in the peak current intensity, calculated as the increment of the signal recorded for PSNP/PEP-modified membranes compared to just PEP-modified membranes divided by the original value (PEP-modified membranes), to evaluate the introduction of the conjugated nanoparticles and select the condition that maximizes the percentage change.

### Electrostatic effects using a negatively charged redox indicator

**Fig. 1:**
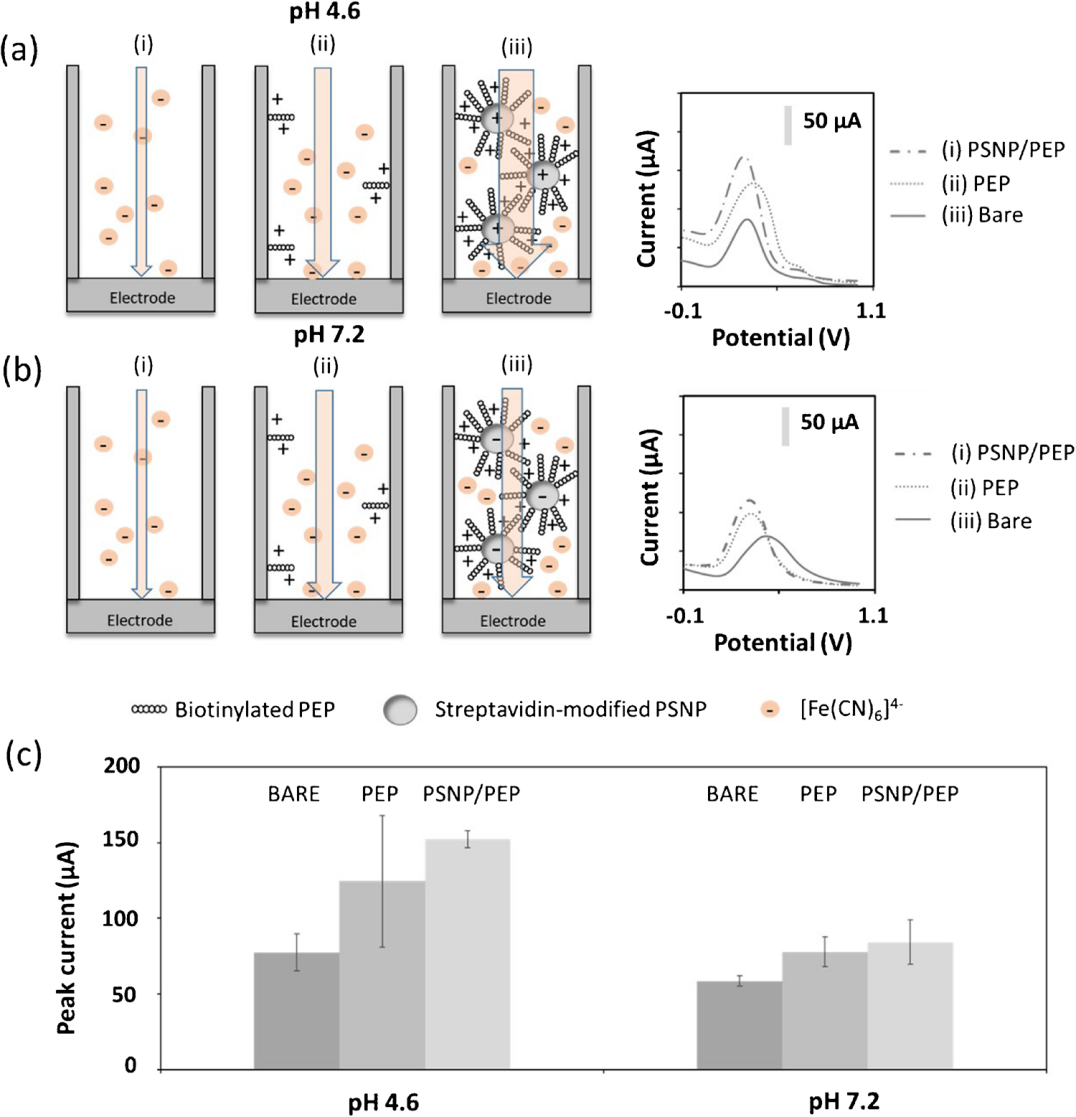
Electrostatic effects involved using [Fe(CN_6_)].^4−^ as redox indicator. Illustration of the phenomena occurring inside the nanochannels, for bare membranes (left) and for membranes modified with PEP (middle) and with PSNP/PEP conjugate (right), together with the corresponding voltammetric signals obtained when the measurements are performed at pH 4.6 (**a**) and at pH 7.2 (**b**). Arrows illustrate the ionic flow velocity through the channels. **c** shows a comparative bar chart of the analytical signals (voltammetric peak current) obtained by triplicate. Measurement buffers: 0.1 M NaAc pH 4.6 and 0.1 M Tris–HCl pH 7.2. Redox indicator solution: 10 mM K_4_[Fe(CN)_6_]. DPV conditions: pre-treatment at − 0.10 V for 30 s; scan from − 0.10 to + 1.10 V; step potential: 0.01 V; modulation amplitude: 0.05; modulation time: 0.01 s; interval time: 0.5; scan rate: 0.02 V/s. Data are given as average ± SD (*n* = 3)

First, the well-known negatively charged redox indicator [Fe(CN_6_)]^4−^ was evaluated in buffer solutions at pH 4.6 and pH 7.2. Working at more basic pH was not considered to avoid damaging the membrane [26]. At pH 4.6 (Fig. 1a) (below the isoelectric point of the PEP), the PEP is positively charged which generates a net positive electrostatic field inside the channels that attracts the negatively charged [Fe(CN_6_)]^4−^ ions. This leads to an increase in the voltammetric signal, compared with that recorded for the bare membrane. This effect is enhanced when the membranes modified with the PSNP/PEP conjugate are evaluated thanks to (i) the amplification effect of PSNP, as a carrier of a big amount of PEP molecules, and (ii) the positive charge of the PSNP themselves, since the streptavidin covering their surface is also positively charged at pH 4.6 (below the isoelectric point of streptavidin). In more detail, the increase in the voltammetric peak current provided by the PSNP/PEP conjugate compared to the PEP condition is about 18% (Fig. 1c).

It is also important to note that the use of PSNP/PEP conjugate seems to improve the reproducibility of the signals, as evidenced by the error bars represented in such a graph. These results could be due to the difference in charge between the PSNP/PEP conjugate and the PEP alone. In the first one, the contribution to the net charge of the streptavidin present in the PSNP surface seems to surpass, under this condition, the impact of the net charge of the PEP. The larger size of this molecule (around 50 kDa) [27] compared to the PEP alone (around 928 Da) and the 3D complexity of this tetrameric protein lead to the fact that the contribution of its charge to the overall electrostatic effects inside the channel masks the contribution of the PEP alone.

However, in the second situation, when the PEP is not conjugated, there are two factors that determine the charge of the molecule: (i) the charges of the individual amino acids that compose the linear peptide, and (ii) the presence of a biotin molecule in the outer end. In the nine amino acid sequence of the target peptide, two of them are charged polar amino acids, arginine and lysine, with pKas of 12 and 10 respectively [28]. The rest of the amino acids are non-polar or polarly uncharged residues that do not contribute to the net charge of the PEP. That means that under that pH value, they are protonated, what leads to the presence of a positive charge per each amino acid, thus two positive charges in the target PEP.

Moreover, it could not be bypassed the fact that the N-terminus α-amino group of the target peptide is biotinylated, and that this protein could also have an effect over the net charge of the PEP, having an isoelectric point of 3.5 [29]. In contrast to streptavidin, biotin is a small-size protein of just about 244 Da, even smaller than the target PEP. This would lead to the fact that, at a pH above 3.5 (as it is the used 4.6 pH), the positive charge of the PEP and the negative charge of the biotin are outcompeting for interaction with the redox indicator, what could explain the deviation observed in this experimental condition. However, the differences observed for pH 4.6 and pH 7.2 could not be clearly explained with the information available. Additional studies on the isoelectric point of the conjugate PEP-biotin would help to elucidate this variation.

This points out the relevance of using PSNP/PEP as a carrier agent, which helps to homogenize the electrostatic effects inside the nanochannels.

**Fig. 2:**
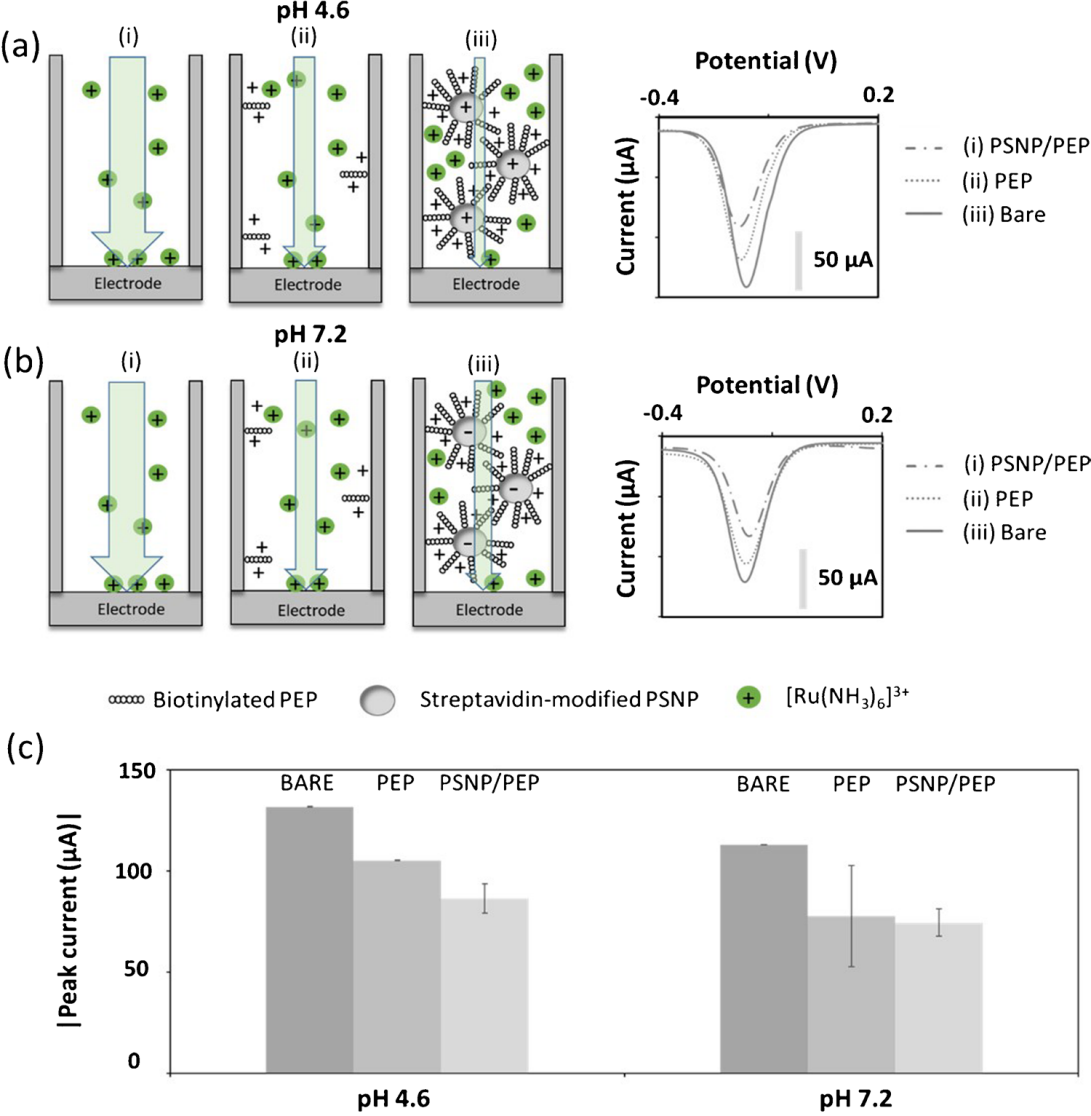
Electrostatic effects involved using [Ru(NH_3_)_6_].^3+^ as redox indicator. Illustration of the phenomena occurring inside the nanochannels, for bare membranes (left) and for membranes modified with PEP (middle) and with PSNP/PEP conjugate (right), together with the corresponding voltammetric signals obtained when the measurements are performed at pH 4.6 (**a**) and at pH 7.2 (**b**). Arrows illustrate the ionic flow velocity through the channels. **c** shows a comparative bar chart of the analytical signals (voltammetric peak current) obtained by triplicate. Measurement buffers: 0.1 M NaAc pH 4.6 and 0.1 M Tris–HCl pH 7.2. Redox indicator solution: 500 µM [Ru(NH_3_)_6_]Cl_3_. DPV conditions: pre-treatment at + 0.20 V for 30 s; scan from + 0.20 to − 0.40 V; step potential: − 0.01 V; modulation amplitude: 0.05; modulation time: 0.01 s; interval time: 0.5; scan rate: 0.02 V/s. Data are given as average ± SD (*n* = 3)

If the measurement solution is adjusted to a pH of 7.2 (Fig. 1b), the peptide is still positively charged, which is reflected in an increase in the voltammetric signal compared with the bare membrane. However, the situation is quite complex in the case of the PSNP/PEP conjugates. At this pH (above the pI of the streptavidin but below the pI of the PEP), the PSNPs are negatively charged, while the PEP molecules in their surface are positively charged. The slight increase in the voltammetric signal obtained suggests that the net charge of the conjugate is positive, which is in line with the results of the ζ potential (Fig. S3) analysis. However, this counteraction of charges minimizes the amplification effect of the PSNP/PEP conjugate (about 7% increase, as defined above) compared with that observed for pH 4.6 (Fig. 2c).

Notably, a difference in the net current obtained at pH 4.6 and pH 7.2 is observed in all the conditions measured. This could be due to the difference in the pH and composition of the measurement buffers used that could affect the current intensity obtained. These slight differences are not significant as comparisons are calculated as the increment in % change for the different conditions studied.

### Electrostatic effects using a positively charged redox indicator

Our findings suggest that positive net charges are present inside the modified nanochannels at a wide range of working pH values. In this context, the evaluation of a positively charged redox indicator is of great relevance to find the optimum conditions for the nanochannel blocking by the PSNP/PEP conjugate.

[Ru(NH_3_)_6_]^3+^ was selected for such a study, which was also performed at pH 4.6 and pH 7.2. In this case, at pH 4.6 (Fig. 2a), the net positive electrostatic field generated by the peptide exerts a repulsion effect to the positively charged [Ru(NH_3_)_6_]^3+^ ions, leading to a reduced flux of ions, evidenced by a decrease in the voltammetric signal compared with that recorded for the bare membrane. This effect is enhanced when the membranes modified with the PSNP/PEP conjugate are evaluated (about 21%, as defined above), due to the amplification effect of the PSNP, as discussed in the case of the [Fe(CN_6_)]^4−^ (Fig. 2c).

Working at pH 7.2 (Fig. 2b), the peptide remains positively charged, observing again a decrease in the voltammetric signal compared with the bare membrane. In the case of the PSNP/PEP conjugate, the negatively charged PSNPs counteract to a large degree the positive charges of the PEP molecules, what is reflected in the low decrease in the voltammetric signal compared with the obtained for the PEP-modified membranes. This behavior highly minimizes the amplification effect of the PSNP/PEP conjugate, being a decrease only of about 4% (Fig. 2c).

Again, the use of PSNP/PEP conjugate increases the reproducibility obtained compared to the membranes modified with PEP alone, what it is in correlation with the above explanation.

As conclusion of this study, the optimum conditions for a maximum blockage of the nanochannels are obtained working with a positively charged redox indicator ([Ru(NH_3_)_6_]^3+^) at a pH 4.6, as it provides for the PSNP/PEP conjugate a decrease of 21% which is slightly higher, in absolute terms, than the one obtained with the negatively charged redox indicator, which generates a decrease in the percentage change of 18%. Such conditions are selected for the detection of MMP-9, whose enzymatic cleavage action should be able to degrade the PSNP/PEP conjugate, increasing the signal recorded.

## Electrochemical detection of MMP-9 by enzymatic cleavage of PSNP/PEP conjugate

**Fig. 3:**
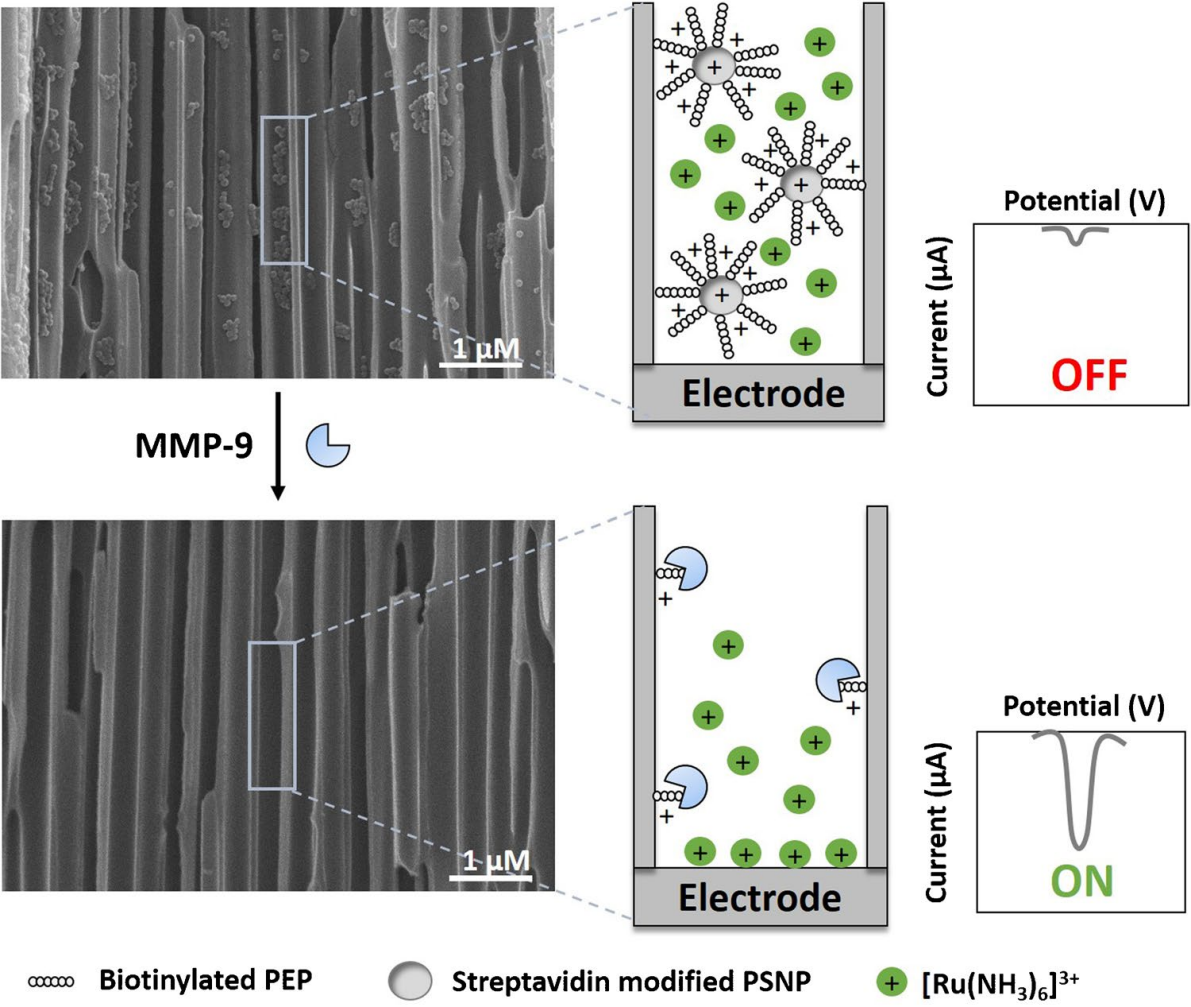
Schematic methodology (not in scale) of the biosensing system based on the blockage of nanochannels by the PSNP/PEP conjugate and further unblockage by enzymatic cleavage of MMP-9. SEM (cross-sectional view) images correspond to the inner walls of the nanoporous alumina membranes with PSNP/PEP immobilized (up) and after the reaction with MMP-9 and a washing step (down)

The MMP-9 sensing approach proposed in this work is schematized in Fig. 3. First, the blockage of the diffusion of [Ru(NH_3_)_6_]^3+^ redox indicator ions to the ITO/PET electrode surface through the nanochannels to the electrode is achieved by the immobilization of the conjugate of PSNP with the peptide Leu-Gly-Arg-Met-Gly-Leu-Pro-Gly-Lys (specific to MMP-9), due to the above discussed electrostatic effects. MMP-9 enzymatic activity cleavages the peptide between methionine (M) and glycine (G) [18], releasing the PSNP that is removed during the cleaning steps, leaving just a small fraction of the peptide immobilized inside the nanochannel. This leads to the unblockage of the nanochannel, allowing an increase in the flux of the re-ox indicator ions through the channels, which is monitored through an increase in the voltammetric signal of reduction of [Ru(NH_3_)_6_]^3+^ to [Ru(NH_3_)_6_]^2+^. SEM images in Fig. 3 also corroborate our hypothesis. The above micrograph shows the presence of the PSNP/PEP conjugate immobilized in the inner walls of the nanochannels (nanoparticles of around 69 nm), conjugates that are removed after the reaction with MMP-9 and a washing step (down micrograph).

**Fig. 4:**
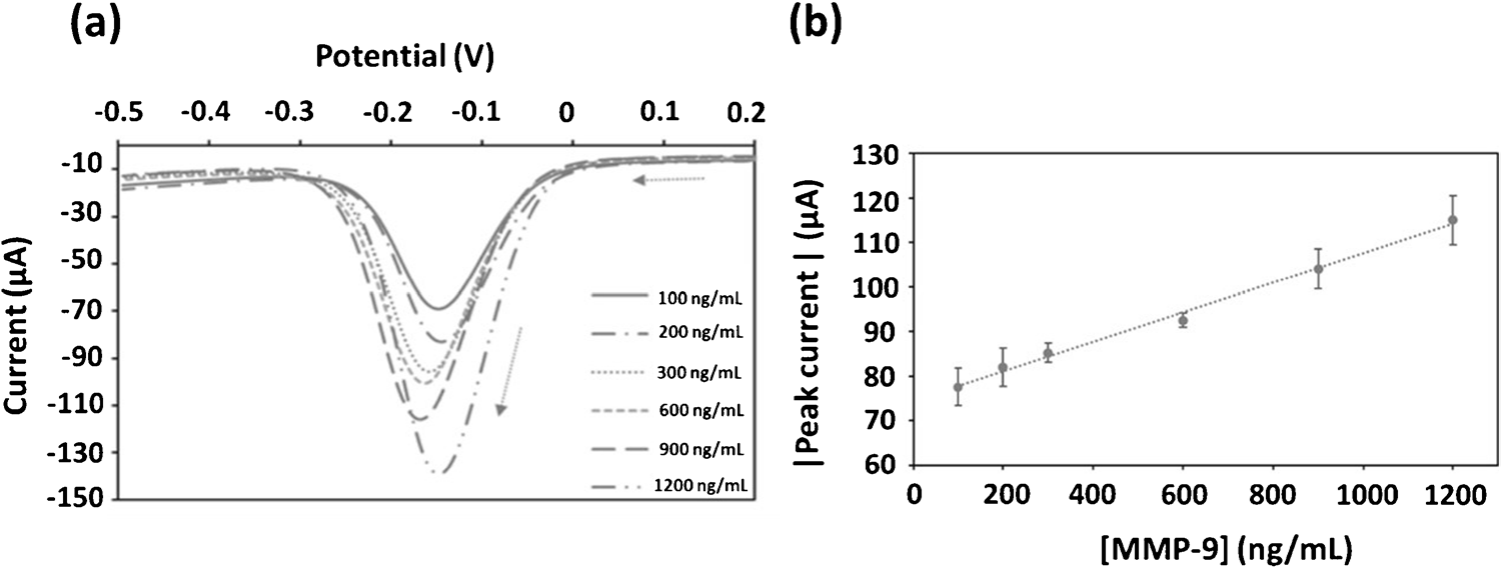
**a** MMP-9 determination through the enzymatic cleavage of Leu-Gly-Arg-Met-Gly-Leu-Pro-Gly-Lys peptide in the PSNP/PEP conjugate, leading to nanochannel unblockage. **a** Differential pulse voltammograms registered in 500 µM [Ru(NH_3_)_6_].^3+^/0.1 M NaAc pH 4.6 after incubation with increasing concentrations of MMP-9 (100–1200 μg/mL, from up to down). DPV conditions: pre-treatment at + 0.20 V for 30 s; scan from + 0.20 to − 0.50 V; step potential: − 0.01 V; modulation amplitude: 0.05; modulation time: 0.01 s; interval time: 0.5; scan rate: 0.02 V/s. **b** Calibration plot obtained for standard solutions of MMP-9. Data are given as average ± SD (*n* = 3)

For the MMP-9 determination, PSNP/PEP-modified membranes were incubated with increasing concentrations of MMP-9 within a linear range of 100–1200 ng·mL^−1^. These values are in accordance with the clinically relevant MMP-9 concentrations found in patients with oral squamous cell carcinoma (OSCC) [30], under the conditions detailed in the “Methods” section. The concentration above and below the linear range of 100–1200 ng·mL^−1^ was tested but did not feat with a linear regression. As shown in Fig. 4a, an increase in the voltammetric signal is observed with the increase in the concentration of MMP-9. Both parameters are adjusted to a linear relationship in the range 100–1200 ng·mL^−1^ (Fig. 4b), with a correlation coefficient of 0.9945, according to the following equation:

$$\left|\mathrm{Peak}\;\mathrm{current}\right|\;\left[{\mu A}\right]\;=\;\left(0.33\;\pm\;0.002\right)\;\left[\mathrm{MMP}-9\right]\;\left(\mathrm{ng}\;\cdot\;\mathrm{mL}^{-1}\right)\;+\;\left(74.577\;\pm\;0.829\right)$$

The method shows good reproducibility, with an intra-day relative standard deviation (RSD) of 8% (*n* = 3). The limit of detection (LOD), calculated as three-time standard deviation of intercept by the slope, is 75 ng·mL^−1^ of MMP-9, while the limit of quantification (LOQ), calculated as ten-time standard deviation of intercept by the slope, is 251 ng·mL^−1^. Hence, the resulting linear range of the developed sensor goes from 251 to 1200 ng·mL^−1^. This value is below the reported as of clinical interest, as shown in the literature for, i.e., the differential diagnosis of oral squamous cell carcinoma (OSCC) [30, 33].

## Selectivity for MMP-9 detection and long-term stability

Selectivity against other biomolecules that can be present in saliva, like uric acid and lysozyme, has been evaluated as possible interference molecules. Both molecules have been evaluated in the same rates in which they are found in real samples, which is 250 μM for uric acid [23] and 50 μg·mL^−1^ for lysozyme [22]. The interference of these molecules has been tested alone, in the presence of MMP-9 at a concentration of 600 ng·mL^−1^ and all three of them together in the appropriate rate.

**Fig. 5:**
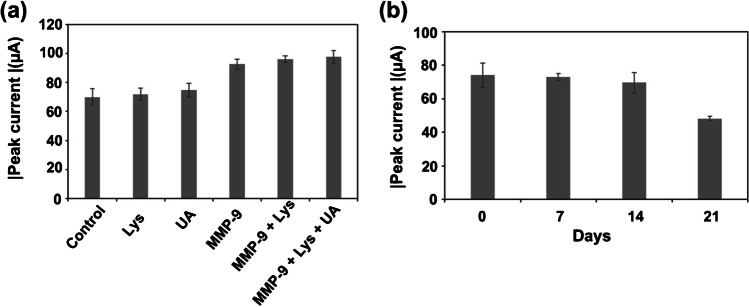
**a** Selectivity evaluation of MMP-9 (concentration: 600 ng·mL^−1^) detection against other possible interferences as lysozyme (Lys) (concentration: 50 µg·mL.^−1^), uric acid (UA) (concentration: 250 µM), alone and in combination with MMP-9. **b** Long-term stability study of the PSNP/PEP-modified membranes. Data are given as average ± SD (*n* = 3)

As shown in Fig. 5a, no significant change in the peak current was observed for any of the assayed proteins, as it is observed for MMP-9, suggesting a good selectivity of our approach. Moreover, the presence of interference molecules did not seem to affect MMP-9 cleavage when combined in the same ratio as they are present in real samples.

The stability of the PSNP/PEP-modified membranes was studied after storing them at 4 °C for a time period of 21 days.

As shown in Fig. 5b, the nanochannel blocking index was stable for the first 2 weeks, noticing a decrease in efficiency after 21 days of storage. Although this aspect requires further optimization, the preliminary stability observed suggests that the PSNP/PEP-modified membranes could be stored ready for the sample analysis, which would only involve the steps of the sample incubation on the membrane, washing and measuring buffer adding, what it would redound in the detection of MMP-9 in around 2 h. The time of analyses is comparable to those reported by other methodologies such as the ELISA technique. Thus, further improvement of the enzymatic cleavage step would be preferable to reach similar values as previously published works [9], what it would mean a comparative advantage over alternative methodologies.

## Detection of MMP-9 in saliva samples: spike and recovery assay

The proposed biosensing platform was evaluated for the detection of MMP-9 in a real complex matrix like saliva. This matrix was selected since the presence of MMP-9 in saliva is clearly indicative of possible diseases such as cancerous pathologies like oral squamous cell carcinoma (OSCC), being proposed as an effective biomarker of such disease [30, 33]. In particular, a concentration of 29.27 ± 0.08 ng·mL^−1^ was reported as mean value for controls, while 511.86 ± 0.12 ng·mL^−1^ was estimated for patients suffering OSCC [30]. Also, it has been demonstrated that MMP-9 levels considerably decrease after surgery, showing it as a promising prognostic marker of OSCC.

For this study, a spike and recovery procedure was performed in a saliva sample. As stated above, saliva samples of healthy donors also contain MMP-9 levels, even though they are lower than the limit of detection reached with this methodology. Therefore, the initial concentration of MMP-9 was determined using an ELISA kit, showing a concentration of 52.37 ± 4.13 ng·mL^−1^. This concentration was considered in the spike and recovery assay when spiking adjusting the initial MMP-9 concentrations added (200, 300, and 600 ng·mL^−1^) in accordance (252, 352, and 652 ng·mL^−1^ respectively). These values correspond to the MMP-9 average concentration that is above and below the positive average value of 511 ng·mL^−1^. An additional MMP-9 value of 200 ng·mL^−1^ was selected, considering that it is near the limit of quantification of the developed method. The concentration of MMP-9 closer to the control values of 29 ng·mL^−1^ was not tested as they are below the LOD of the method. It is worth mentioning that, due to the complexity of the matrix and the presence of gelling proteins as mucins, further cleaning steps (6 washing additions instead of 3) were required compared to the use of buffer solutions to eliminate all non-bonded proteins. However, no further treatment was required, as nanoporous membrane act as effective filters. The analytical signals obtained were compared with those reached when dissolving MMP-9 in Tris–HCl buffer, obtaining quantitative recoveries of 110, 81, and 114%, for 252, 352, and 652 ng·mL^−1^ respectively (Table 1). These results demonstrate that the method can differentiate concentrations of MMP-9 in complex samples near the positive average value for OSCC, thus confirming the suitability of the developed sensor for the detection of this biomarker in a real scenario.

**Table 1:** Spike and recovery assay data in saliva for MMP-9 concentrations of 252, 352, and 652 ng·mL^−1^. Data are given as average ± SD (*n* = 3)

Sample	Spiked MMP-9 (ng·mL^−1^)	MMP-9 concentration (ng·mL^−1^)	Current saliva samples (μA)	Calculated MMP-9 concentration (ng·mL^−1^)	Recovery (%)
Saliva from healthy patient	200	252	83.70 ± 1.84	276.5 ± 55.82	110
	300	352	83.97 ± 2.10	284.66 ± 63.25	81
	600	652	99.07 ± 3.91	741 ± 118	114

These results demonstrate that low matrix effects are affecting our methodology when analyzing saliva samples, as well as the effectiveness of the system to potentially discriminate between MMP-9 levels of OSCC patients and healthy controls in such samples.

The methodology showed in this work has advantages versus other previously reported analytical systems (Table 2), especially regarding the sample pre-treatment and the use of labels required. The impressive filtering properties of nanoporous alumina membranes, together with the characteristics of the electrochemical measurements, convert them into ideal sensing platforms for the evaluation of analytes in this case in saliva, independently of the turbidity of the sample and the presence of interference compounds. This absence of sample pre-treatment together with the no need for neither labels nor competitive assays is the main advantage of the developed analytical system. In contrast, the main limitation is related to the LOD, which is quite higher than that of other reported methodologies. Even so, it is low enough for detecting MMP-9 above the positive average value for OSCC, being especially suitable for decentralized screening in healthcare centers.

**Table 2:** An overview of recently reported nanomaterial-based electrochemical methods for the determination of MMP-9 in saliva samples

Sensing principle	Transduction technique	LOD (ng·mL^−1^)	LR (ng·mL^−1^)	Sample pre-treatment	Ref
Fiber-optic and antibody-based microarray platform	Epi-fluorescence	1.31	1.31 − 800	Centrifugation dilution	[34]
Integrated platform using microsphere-based array	Fluorescence	8.62	5.12–500	Centrifugation	[35]
Carboxymethyldextran hydrogel immunosensor chip	Surface plasmon resonance (SPR)	0.008	10–200	Filtering through 0.22 µM membranes	[36]
ELISA	Colorimetric	0.10	0.16–10	Freezing, thawing, and centrifugation	[37]
Nanochannels	Electrochemistry	75	100–1200	No	This work

## Conclusions

Polystyrene nanoparticles have been shown as effective carriers of the peptide Leu-Gly-Arg-Met-Gly-Leu-Pro-Gly-Lys, being able to significantly enhance the electrostatic effects involved in the nanochannel blockage produced when the PEP is immobilized in nanoporous alumina membranes. Interestingly, the different isoelectric points of the PEP and the PSNP (through its streptavidin coating) allow tuning the charge of the electric field generated inside the nanochannels. In our case, working at pH 4.6 was found as optimum so as to have both PEP and PSNP positively charged, generating a strong positive electric field inside the channels. This positive electric field favors the diffusion of negatively charged redox indicator ions ([Fe(CN)_6_]^4−^) while hinders the flow of positively charged ones ([Ru(NH_3_)_6_]^3+^), what is monitored through voltammetric measurements. The hindering in the diffusion of the positively charged ions gave us the maximum discrimination between the bare and PSNP/PEP-modified membranes in terms of voltammetric signal. These conditions were selected as optimum for the detection of MMP-9, whose enzymatic cleavage action is able to degrade the PSNP/PEP conjugate and unblock the nanochannels.

Our method reached a MMP-9 detection limit of 75 ng·mL^−1^, which is low enough for diagnostics applications like the differential diagnosis of oral squamous cell carcinoma (OSCC), for which the excellent performance observed in saliva samples is highly promising. Our approach represents a cheap and fast sensing methodology of great potential in point-of-care diagnostics. However, a subject of study and further improvement is the integrability of the electrochemical cell set-up within nanoporous membranes and working electrodes, being now independent pieces, thus reducing manipulation steps.

Overall, our findings suggest that PSNP may be used as effective enhancers of nanochannel blocking, also offering high versatility in terms of nanoparticle size and charge that may be modulated by the measurement pH. This may be extended not only for the detection of enzymes through enzymatic cleavage processes but also for the detection of other biomarkers, in approaches where the PSNP may be used as tags.

## Supplementary Information

Below is the link to the electronic supplementary material.

Supplementary file1 (DOCX 2132 KB)

## Data Availability

Data will be made available on request.
