# Supplementary material for: Electrochemical monitoring of enzymatic cleavage in nanochannels with nanoparticle-based enhancement: determination of MMP-9 biomarker
Source: Mikrochim Acta. 2023 Jun 12;190(7):257. doi: 10.1007/s00604-023-05835-7 (PMC10258169; doi:10.1007/s00604-023-05835-7)
Supplement: Supplementary file 1 — Supplementary file1 (DOCX 2132 KB) [file 604_2023_5835_MOESM1_ESM.docx]

Supplementary Information

**Electrochemical monitoring of enzymatic cleavage in nanochannels with nanoparticle-based enhancement: determination of MMP-9 biomarker**

David Valero-Calvo^1,2^, Celia Toyos-Rodriguez^1,2^, Francisco Javier García-Alonso^2,3^ and Alfredo de la Escosura-Muñiz^1,2*^

^1^ NanoBioAnalysis Group-Department of Physical and Analytical Chemistry, University of Oviedo, Julián Claveria 8, 33006 Oviedo, Spain

^2^ Biotechnology Institute of Asturias, University of Oviedo, Santiago Gascon Building, 33006 Oviedo, Spain

^3^ NanoBioAnalysis Group-Department of Organic and Inorganic Chemistry, University of Oviedo, Julián Clavería 8, 33006 Oviedo, Spain

*Corresponding author: alfredo.escosura@uniovi.es

**Methods**

**Nanoporous alumina membranes silanization**

Membranes were boiled for 1 h in milli-Q water for both cleaning them and activate the hydroxyl groups present in the surface, what favors the latter silanization process. After drying the membranes using nitrogen, the membranes were immersed in a 5% APTES solution in acetone for 1 h. After washing (3X) with acetone, membranes were baked for 30 min at 120ºC and stored at room temperature until use.

**Collection of saliva sample**

Whole saliva was collected from a healthy volunteer in the early morning before eating, drinking nor brushing teeth to avoid external contamination of the sample matrix. While seated and relaxed, volunteer spat and collected the sample in a 10 mL polypropylene tube being transferred to a 1.5 mL Eppendorf for easier handling. Storage of the sample was performed at -20°C into the freezer.

**Electrochemical measurements**

Measurements were performed using a home-made designed electrochemical set-up (**Fig. S2)**. Nanoporous alumina membranes were placed (with the filtering side up) on top of a piece of ITO-PET of 4.3 x 2 cm and introduced into a methacrylate cell with a hole of 0.8 cm in diameter defining the working electrode. An insulating O-ring was employed to avoid liquid leakage. Platinum wire and silver/silver chloride were used as counter and reference electrodes, respectively.

[Ru(NH_3_)_6_]^3+^ was measured by DPV with a pre-treatment at +0.20 V applied for 30 s followed by a scan from +0.20 V to -0.40 V (Step potential: -0.01 V; Modulation amplitude: 0.05 V; Modulation time: 0.01 s; Interval time: 0.5 s; Scan rate: 0.02 V/s).

In the case of the evaluation of the [Fe(CN)_6_]^4-^, a pre-treatment at -0.10 V was applied for 30 s, followed by a differential pulse voltammetric (DPV) from -0.10 V to +1.10 V (Step potential: 0.01 V, Modulation amplitude: 0.05; Modulation time: 0.01 s; Interval time: 0.5; Scan rate: 0.02 V/s). The anodic peak due to the oxidation of [Fe(CN)_6_]^4-^ ions to [Fe(CN)_6_]^3-^ recorded at approx. +0.40 V represents the analytical signal.

All the measurements were done at room air and temperature. Each membrane was evaluated using a new ITO/PET working electrode.

**Characterization of the PSNP/PEP conjugate**

PSNPs with a nominal diameter of 90 nm were used as carriers of the peptide substrate Leu-Gly-Arg-Met-Gly-Leu-Pro-Gly-Lys, with the aim of maximizing the blocking of the nanochannels, which will be then unblocked by the enzymatic cleavage action of the MMP-9.

In particular, streptavidin-modified PSNP were conjugated with biotinylated PEP (biotin introduced in the N-terminal) following the experimental procedure described at *Methods* section, taking advantage of the affinity linkage biotin-streptavidin, one of the strongest non-covalent bonds in nature [1].

Both PSNP and PSNP/PEP were characterized by TEM (**Fig. S3a**) showing a spherical shape and an appropriate polydispersity. The corresponding histograms give an average mean diameter of 67 ± 6 nm for the PSNP and 69 ± 8 nm for the PSNP/PEP (**Fig. S3b**).

The success of the conjugation process as well as the charge of the nanoparticle suspensions obtained were also confirmed by measuring the ζ-potential, which indicates the surface electrical potential of the analyzed particles (measurements performed in 0.1M Tris-HCl pH 7.2). As shown in **Fig. S3c** unconjugated PSNP show a ζ-potential of -14.7 ± 5.13 mV, being such negative value related to the presence of streptavidin (isoelectric point: 5-6; pH 7.2 is above the isoelectric point of streptavidin) in the PSNP surface. Interestingly, the presence of the positively charged peptide (isoelectric point: 11; pH 7.2 is below the isoelectric point of the peptide) in the PSNP/PEP conjugate makes this value to shift toward positive values (6.26 ± 3.86 mV).

Finally, both PSNP and PSNP/PEP were also characterized by Fourier Transfrom Infrared (FTIR) spectroscopy. Their spectra are shown in **Fig. S3d**, where the ATR IR spectrum of PEP is also included for comparative purposes. The spectrum of the PSNP/PEP (iii) is almost coincident with that one corresponding to the PSNP (ii), as it could be anticipated. However, a closer inspection of the PSNP/PEP spectrum (iii) indicates that the band at about 1600 cm^-1^ is broader (in the upper part) than the same absorption in the PSNP spectrum (ii) due to the presence of the characteristic weak absorption of secondary amides from the amino acids at 1530 cm^-1^, a band clearly observed in the spectrum of PEP (i). Moreover, the curve between the absorptions at 1100 and 1360 cm^-1^ is flatter in (iii) than in (ii) because the spectrum below includes the band a 1200 cm^-1^ present in the PEP spectrum (i). Therefore, the infrared spectra analysis is consistent with the success of the conjugation process.

**Membranes nanopore size optimization**

Nanoporous alumina membranes are commercially available in different pore sizes: 20, 100 and 200 nm. However, as the size of PSNP is bigger than 20 nm, only 100 and 200 nm pore sizes were evaluated. For the study, a PSNP/PEP concentration of 0.05 µg/mL and [Ru(NH_3_)_6_]^3+^ as red-ox indicator in a working buffer of 0.1M pH 4.6 NaAc were employed in order to have the higher electrostatic blockage possible.

Theoretically, it is expected that an increase in the pore size facilitates the flux of ions through the nanochannels with the consequent reduction in the blockage obtained. However, as schematized in **Fig. S5a**, a higher increment in the percentage change, calculated as the increment of the signal recorded for PSNP/PEP modified membranes compared to bare membranes divided by the bare membranes, is obtained when working with 200 nm nanopore (46%) compared with when working with the 100 nm nanopores (17%). This can be due to the fact that the bioconjugate can form small aggregates, hence many of them are not penetrating the nanochannels with pore sizes of 100 nm. In view of these results, membranes containing pores of 200 nm are selected for our sensing system.

**PSNP/PEP incubation time optimization**

The immobilization of the PSNP/PEP conjugate highly depends on the incubation time used. To maximize immobilization, nanoporous alumina membranes were incubated with the PSNP/PEP conjugate for 2 hours and overnight, taking as reference our findings in previous works for the immobilization of other biomolecules (see references [8] and [9] in the main text) . As shown in the **Fig. S5b**, a considerably higher blockage was achieved with an overnight incubation, showing an increase in the percentage change (calculated as the increment of the signal recorded for PSNP/PEP modified membranes compared to bare membranes divided by the bare membranes) from 15% to 54%, when compared with the 2 hours incubation. In view of these results, an overnight incubation is selected for our sensing system.


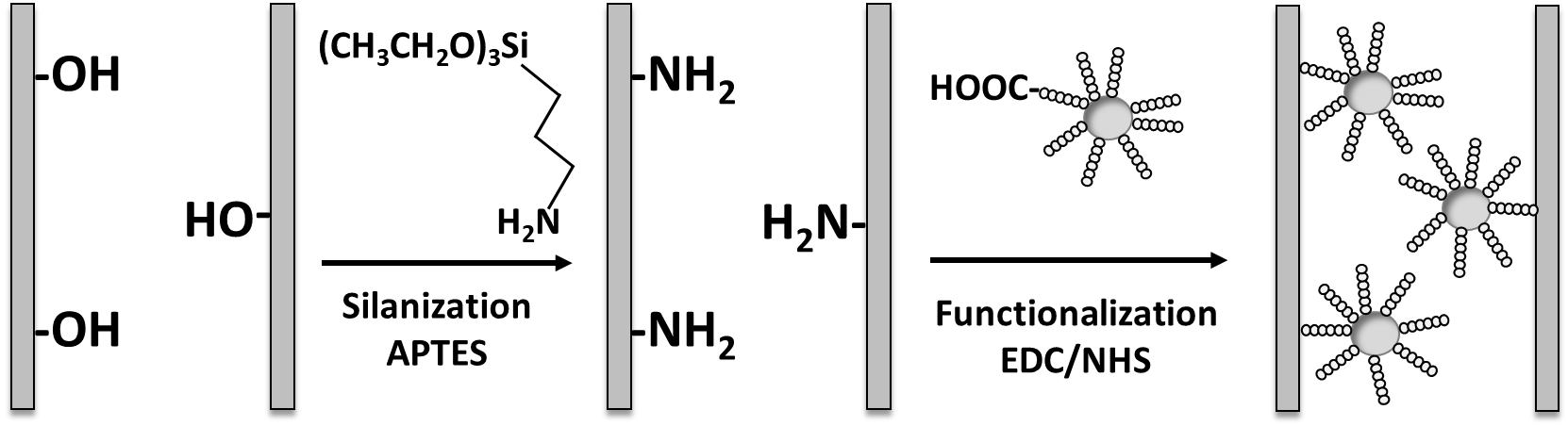


**Fig. S1.** Schematic illustration of the experimental procedure followed for the alumina nanoporous membranes silanization and PSNP/PEP immobilization through the peptide bond.


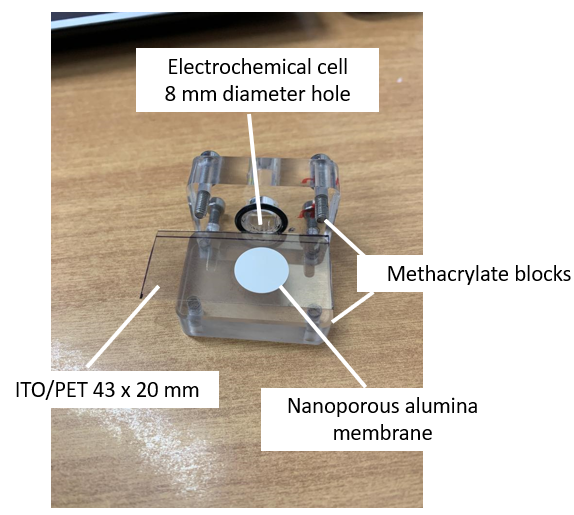

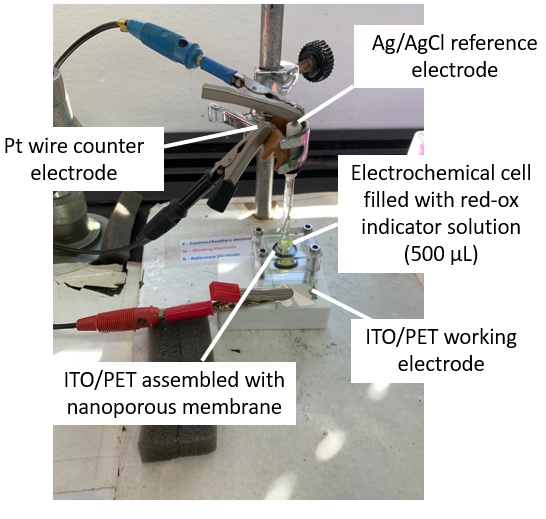


**Fig. S2.** Electrochemical cell set-up.


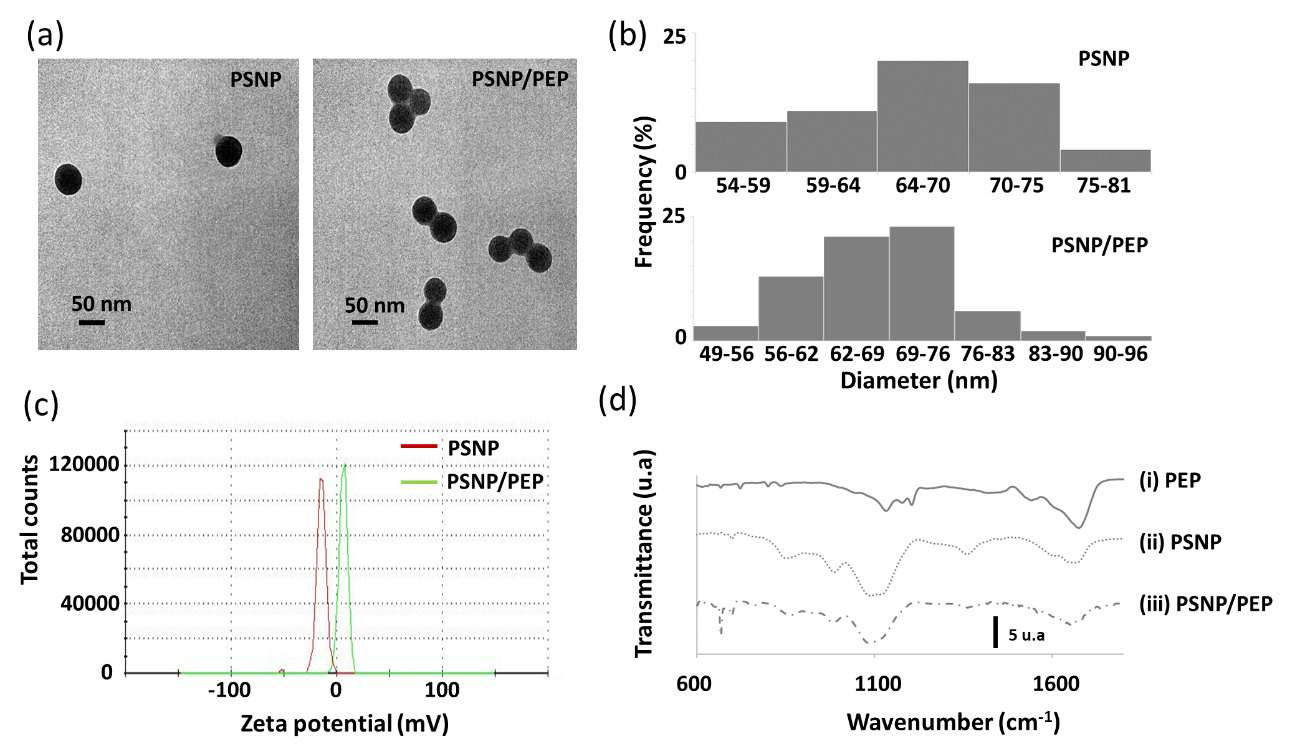


**Fig. S3.** Characterization of PSNP/PEP conjugate. **(a)** TEM images of PSNP (left) and PSNP/PEP conjugate (right). **(b)** Corresponding size distribution histograms for PSNP (up) and PSNP/PEP conjugate (down). **(c)** ζ-potential diagram of PSNP (red line) and PSNP/PEP conjugate (green line). **(d)** FTIR spectra of PEP, PSNP and PSNP/PEP conjugate.


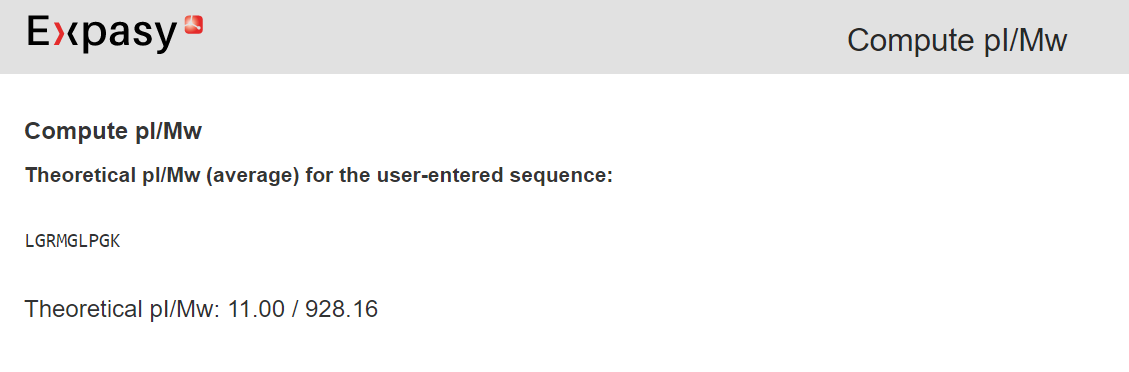
**Fig*.* S4.** Theoretical pI of the peptide substrate (Leu-Gly-Arg-Met-Gly-Leu-Pro-Gly-Lys) calculated by the bioinformatic software Expasy from the sequence of amino acids.


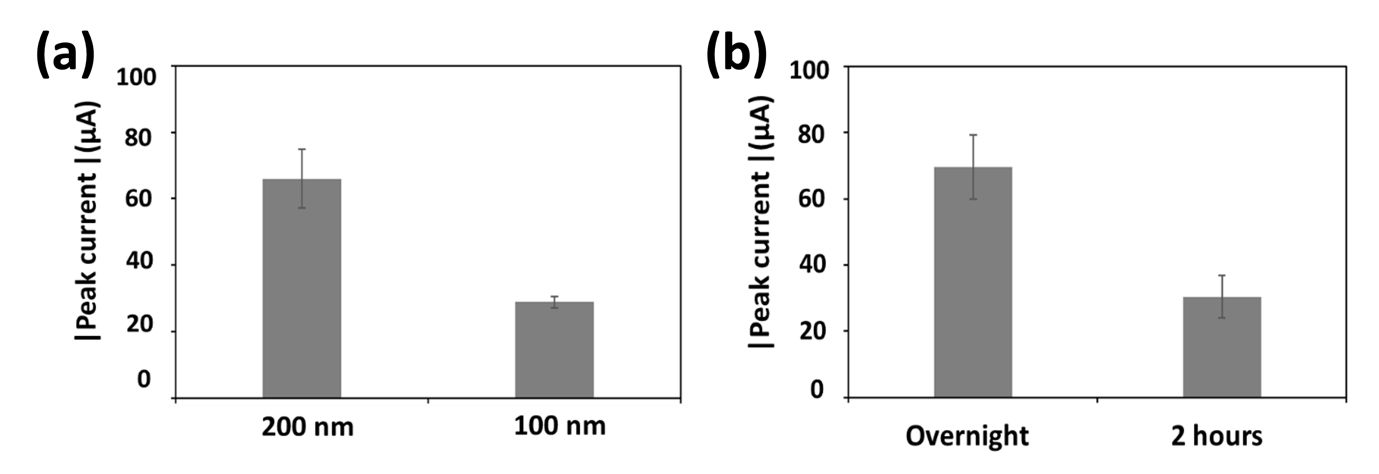


**Fig. S5.** **(a)** Comparative bar graph of the peak current obtained for nanoporous alumina membranes with different pore sizes (100 and 200 nm), modified with PSNP/PEP conjugate **(b)** Comparative bar graph of the peak current obtained for nanoporous alumina membranes of 100 nm modified with PSNP/PEP conjugate over different incubation times (overnight and 2h). The results obtained with both experiments confirm that 200 nm and overnight incubation are the optimum conditions for PSNP/PEP conjugation, as it has been used along the text.

**References**

1. Liu F, Zhang JZH, Mei Y (2016) The origin of the cooperativity in the streptavidin-biotin system: A computational investigation through molecular dynamics simulations. Sci Rep 2016 6:27190. https://doi.org/10.1038/srep27190
